# Carriage of antibiotic resistance genes to treatments for chlamydial disease in koalas (*Phascolarctos cinereus*): A comparison of occurrence before and during catastrophic wildfires

**DOI:** 10.1016/j.onehlt.2023.100652

**Published:** 2023-11-10

**Authors:** Fiona K. McDougall, Wayne S.J. Boardman, Natasha Speight, Tamsyn Stephenson, Oliver Funnell, Ian Smith, Petra L. Graham, Michelle L. Power

**Affiliations:** a School of Natural Sciences, Faculty of Science and Engineering, Macquarie University, Sydney, NSW 2109, Australia; b School of Animal and Veterinary Sciences, Faculty of Sciences, Engineering and Technology, University of Adelaide, Roseworthy, SA 5371, Australia; c Zoos South Australia, Frome Rd, Adelaide, SA 5001, Australia; d School of Mathematical and Physical Sciences, Faculty of Science and Engineering, Macquarie University, Sydney, NSW 2109, Australia

**Keywords:** Antimicrobial resistance, *Chlamydia pecorum*, Chlamydiosis, One health, Bushfires, Koala conservation

## Abstract

Growing reports of diverse antibiotic resistance genes in wildlife species around the world symbolises the extent of this global One Health issue. The health of wildlife is threatened by antimicrobial resistance in situations where wildlife species develop disease and require antibiotics. Chlamydial disease is a key threat for koalas in Australia, with infected koalas frequently entering wildlife hospitals and requiring antibiotic therapy, typically with chloramphenicol or doxycycline. This study investigated the occurrence and diversity of target chloramphenicol and doxycycline resistance genes (*cat* and *tet* respectively) in koala urogenital and faecal microbiomes. DNA was extracted from 394 urogenital swabs and 91 faecal swabs collected from koalas in mainland Australia and on Kangaroo Island (KI) located 14 km off the mainland, before (*n* = 145) and during (*n* = 340) the 2019–2020 wildfires. PCR screening and DNA sequencing determined 9.9% of samples (95%CI: 7.5% to 12.9%) carried *cat* and/or *tet* genes, with the highest frequency in fire-affected KI koalas (16.8%) and the lowest in wild KI koalas sampled prior to fires (6.5%). The diversity of *cat* and *tet* was greater in fire-affected koalas (seven variants detected), compared to pre-fire koalas (two variants detected). Fire-affected koalas in care that received antibiotics had a significantly higher proportion (*p* < 0.05) of *cat* and/or *tet* genes (37.5%) compared to koalas that did not receive antibiotics (9.8%). Of the *cat* and/or *tet* positive mainland koalas, 50.0% were *Chlamydia*-positive by qPCR test. Chloramphenicol and doxycycline resistance genes in koala microbiomes may contribute to negative treatment outcomes for koalas receiving anti-chlamydial antibiotics. Thus a secondary outcome of wildfires is increased risk of acquisition of *cat* and *tet* genes in fire-affected koalas that enter care, potentially exacerbating the already significant threat of chlamydial disease on Australia's koalas. This study highlights the importance of considering impacts to wildlife health within the One Health approach to AMR and identifies a need for greater understanding of AMR ecology in wildlife.

## Highlights

• Resistance genes to doxycycline (*tet*) and chloramphenicol (*cat*) found in 9.9% koalas.
• Fire-affected koalas had the highest frequency of *cat* and/or *tet* genes (16.8%).
• *cat* and *tet* gene diversity was greater in fire-affected koalas compared to pre-fire.
• 50% of *cat/tet* positive mainland Australia koalas tested positive for *Chlamydia.*
• Presence of *cat/tet* may contribute to negative chlamydiosis treatment outcomes.

## 1 Introduction

Antimicrobial resistant bacteria have now spread beyond humans and domestic animals to the environment and the wildlife within [1]. Consequently, antimicrobial resistance (AMR) must be addressed using a One Health approach, that recognises the interconnected relationships of human, animal and environmental health [2]. However, current AMR One Health approaches are typically anthropo-centric, with the focus being on the role of wildlife as reservoirs of antimicrobial resistant bacteria and the associated zoonotic risks, and a failure to consider the impacts of AMR to wildlife health and conservation [3,4]. Increasing reports of antimicrobial resistant bacteria in wildlife poses a growing risk to wildlife health, especially given resistant strains are frequently pathogenic for a range of host species [[5], [6], [7], [8], [9], [10], [11]]. The threat associated with AMR is more pronounced when an infectious disease threatens a wildlife species and antibiotics become a main tool for species conservation [12].

The koala (*Phascolarctos cinereus*), an iconic marsupial species endemic to Australia, is impacted by chlamydiosis, an infectious disease identified as having a high risk to koala population viability [13]. The causal pathogen of koala chlamydial disease is typically *Chlamydia pecorum*, a gram-negative intracellular bacterial species [14]. The prevalence of chlamydial disease in koalas varies across geographical and temporal scales, ranging from 0% to 71% across the koalas range in Australia [12]. At present, koalas on Kangaroo Island (KI), located 14 km off the coast of mainland South Australia (SA), appear to be the largest remaining *Chlamydia*-free population [15]. Whereas koalas in mainland SA have a reported prevalence of chlamydial disease ranging from 4% to 63% [[15], [16], [17]].

Clinical disease from *C. pecorum* in koalas most commonly manifests as cystitis, reproductive tract disease, renal disease and/or conjunctivitis, with advanced disease causing infertility and/or blindness [14]. Koalas with urogenital and ocular chlamydial disease frequently require antibiotic therapy [18], but antibiotic treatment options are extremely limited due to sensitivity of the koala gut microbiome [19]. Antibiotic administration may induce composition shifts in the koala gut microbiome, including a reduced abundance of specialised gut bacteria essential for digesting eucalypt leaves [19,20]. Subsequently, this shift can lead to loss of the mucobacterial lining of the caecum and proximal colon, which frequently leads to emaciation and death, a syndrome referred to as fatal gastrointestinal dysbiosis [19]. Only two antibiotics, chloramphenicol and long-acting doxycycline (tetracycline class of antibiotics), are currently considered to be the anti-chlamydial treatments of choice [18,[21], [22], [23], [24]]. In addition to infectious agents, koalas are subject to numerous key threatening processes that are driving variable rates of decline across their range in Australia, including habitat loss, wildfires, climate change, dog attacks and vehicle collisions [25]. As such, koalas frequently enter wildlife hospitals and rehabilitation centres, requiring veterinary treatment which often involves antibiotic administration [26,27].

In wildlife hospitals and other captive environments, animals typically have a higher prevalence of antimicrobial resistant bacteria compared to their free-living counterparts [10,28]. The higher carriage of AMR in the microbiomes of captive wildlife has been associated with several factors including, higher levels of exposure to sources of resistant bacteria, increased transmission of resistant bacteria between captive wildlife, and selective pressures associated with administration of antibiotics in captive settings [10,[29], [30], [31]]. The frequent use of chloramphenicol and doxycycline for treatment of chlamydiosis in koalas has potential to select for resistance genes to these antibiotics [32].

Resistance genes to chloramphenicol and doxycycline have been reported in a diverse range of bacterial species including many pathogenic strains [32]. Over 35 chloramphenicol resistance genes have been identified, with the majority (21 of 35) encoding chloramphenicol *O*-acetyltransferase (*cat*) enzymes [32]. The *cat* variants *catA1, catA2, catA3* and *catA4* have been reported in over 30 genera of predominantly gram-negative and some gram-positive bacteria [32]. Over 40 genes conferring resistance to tetracyclines have been identified, of which 29 genes encode efflux proteins that are able to export tetracyclines out of bacterial cells [32]. The most frequently observed tetracycline efflux genes in gram-negative bacteria belong to the *tet* family, with *tet*(A), *tet*(B), *tet*(C), *tet*(D), *tet*(E) and *tet*(G) each reported in between 11 and 33 gram-negative bacterial genera [32]. Antibiotic resistance has however been rarely observed in *Chlamydia* spp. [33], with the exception of some *Chlamydia suis* strains, a species primarily infecting pigs, which have acquired a *tet*(C) gene from a non-chlamydial bacterial strain via horizontal gene transfer [34].

Given the importance of chloramphenicol and doxycycline for treating chlamydial disease in koalas, this study aimed to investigate the occurrence and diversity of target chloramphenicol and doxycycline resistance genes in urogenital and faecal microbiomes. During the summer of 2019–2020 koalas were impacted by catastrophic wildfires that caused destruction of almost 12.6 million hectares of forest, much of which was koala habitat, and the loss of thousands of koalas [[35], [36], [37]]. Hundreds of fire-affected koalas were rescued with many requiring veterinary treatment for fire-related injuries and/or chlamydial disease and periods of rehabilitation in wildlife care settings [[38], [39], [40]]. A second aim of this study was to compare the dynamics of chloramphenicol and doxycycline resistance between fire-affected koalas receiving veterinary treatment and non-fire-affected koalas.

## 2 Materials and methods

### 2.1 Conservation and distribution of koalas (*Phascolarctos cinereus*)

**Fig. 1:**
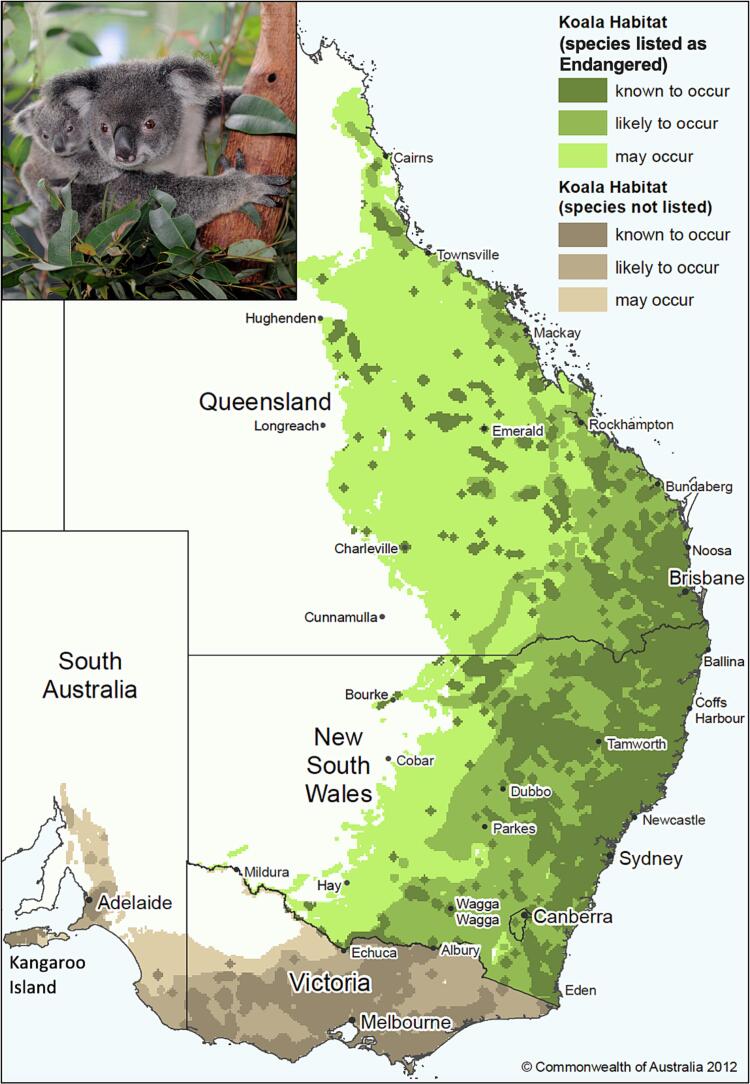
Map showing the distribution of koalas in Australia. Green shaded areas indicate regions in which koalas are listed as endangered and brown shaded areas indicates regions where koalas are not listed as threatened. Map image modified under a Creative Commons (CC) Attribution 4.0 International licence [39]. Koala image sourced from Shutterstock under a standard licence (wilsmedia, Stock Photo ID: 1169355835). (For interpretation of the references to colour in this figure legend, the reader is referred to the web version of this article.)

The koala is an arboreal marsupial belonging to the Family Phascolarctidae. They inhabit coastal areas spanning four Australian states; Queensland, New South Wales, Victoria and SA, and occasionally the Australian Capital Territory (Fig. 1). The conservation status of koalas is highly variable. In the northern areas of eastern Australia koalas are in decline, and the species is currently listed as endangered in Queensland, New South Wales, and the Australian Capital Territory under the Environment Protection and Biodiversity Conservation Act [41] (Fig. 1). In contrast, koalas in the southern states of Australia, Victoria and SA, are not undergoing decline, and consequently, not listed as threatened in these states or nationally [41] (Fig. 1).

### 2.2 Sample sources

DNA samples derived from faecal swabs, urogenital swabs or chlamydial swabs were used in this study. Faecal swabs (*n =* 91) were opportunistically collected as part of a wild koala health surveillance project from koalas in three national parks or reserves within the Mount Lofty Ranges (MLR) in SA (Table 1). DNA was extracted as part of this study using the ISOLATE II Fecal DNA Kit (Bioline, London, UK) according to manufacturer's instructions.

**Table 1:** Sources of DNA samples used in this study and chlamydial diagnostic qPCR testing method references. MLR, Mount Lofty Ranges; SA, South Australia.

Swab type	Collection location	Collection date	No. of DNA samples	No. 16S PCR positive DNA samples^a^	Wild-caught / In-care	Fire-affected / Non-fire-affected	Chlamydial qPCR method references
Faecal	Cleland, MLR	April 2018	39	39	Wild	Non-fire	Not tested
	Belair, MLR	April 2018	29	29	Wild	Non-fire	Not tested
	Morialta, MLR	April 2018	23	23	Wild	Non-fire	Not tested
Urogenital	Kangaroo Island	2014–2017	49	46	Wild	Non-fire	[15]
		2019–2020	111	95	In-care	Fire	[43,44]
Chlamydial	Mainland Australia	2019–2020	11	8	In-care	Non-fire	[45]
		2019–2020	272	245	In-care	Fire	[45]
Total			534	485			

a Only 16S PCR positive samples were included in this study.

DNA samples derived from urogenital swabs (cloaca/urogenital sinus in female koalas and from the penile urethra in male koalas) or chlamydial swabs (combined urogenital and conjunctival swabs) were provided for this study by multiple sources (Table 1). All urogenital and chlamydial swabs were collected for routine chlamydial disease diagnostic qPCR testing and provided as extracted DNA (Table 1). Urogenital swab samples from Kangaroo Island (KI), SA (*n =* 160) were collected as part of the Koala Sterilisation Program (49 of 160) [15] and from koalas receiving veterinary care during the 2019–2020 bushfire season (111 of 160) [38]. Chlamydial swab DNA samples from Mainland Australia (mAUS) were collected from koalas in veterinary care during 2019–2020 (*n =* 283), with the majority being fire-affected (272 of 283), and provided by the Asia Pacific Centre for Animal Health (APCAH) at The University of Melbourne, Victoria (Table 1)*.*

Where available, samples were accompanied by information or data was extracted from provided animal treatment records. Data included fire season status, koala location, koala sex, history of antibiotic administration, chlamydial disease clinical signs scores and diagnostic qPCR results (Supplementary Data 1).

### 2.3 16S rRNA PCR screening of DNA samples

Prior to antibiotic resistance gene screening all DNA samples (*n* = 534) underwent a 16S rRNA PCR to confirm competency and suitability for inclusion in further analyses. The 16S PCRs used the universal eubacterial primers f27 and r1492 [42] with GoTaq® Green Master Mix (Promega, Madison, USA) and cycling conditions of 94 °C 3 min; 35 cycles of 94 °C 30 s, 58 °C 30 s, 72 °C 1 min 30 s; 72 °C 5 min. Samples which failed to amplify in the 16S rRNA PCR were excluded from further analysis (Table 1).

### 2.4 PCR screening for chloramphenicol and doxycycline resistance genes

Two multiplex PCRs were utilised to screen for antibiotic resistance genes; the first to detect four *cat* gene variants which confer chloramphenicol resistance, namely *catA1, catA2, catA3* and *catA4*) [46], and the second to detect six variants which confer tetracycline/doxycycline resistance, namely *tet*(A), *tet*(B), *tet*(C), *tet*(D), *tet*(E) and *tet*(G) [47]. The *cat* multiplex PCR used four forward primers targeting specific *cat* genes and one generic reverse primer (Supplementary Table S1) [46]. The *tet* multiplex PCR used one generic forward primer and six reverse primers targeting the six specific *tet* genes (Supplementary Table S1) [47]. All primer concentrations were 0.5 μM and 3 μl DNA was used per 20 μl reaction. Both multiplex PCRs were performed using AllTaq Master Mix (Qiagen, Hilden, Germany) and the following cycling conditions; *cat* multiplex, 95 °C 3 min; 40 cycles of 95 °C 15 s, 50 °C 30 s, 72 °C 1 min; 72 °C 5 min, and *tet* multiplex, 95 °C 3 min; 40 cycles of 95 °C 15 s, 55 °C 30 s, 72 °C 1 min; 72 °C 5 min. Positive control DNA for *catA1, catA2, tet*(A), *tet*(B), *tet*(C) and *tet*(D) were included in the respective multiplex PCRs and two negative controls were included in each PCR; PCR grade H_2_O and *Chlamydia suis* DNA (strain S45, tetracycline susceptible) [48]. Samples that were presumptively positive for the *catA1* gene were screened in a single gene PCR using UCP Multiplex Master Mix (Qiagen, Hilden, Germany) with primers CAT-1 and CAT-R under the same conditions as the multiplex *cat* PCR, to confirm the presence of a *catA1–1* or *catA1–2* gene variant. All PCR products were visualised by gel electrophoresis using a 2% agarose gel, 1 x TBE buffer and SYBR™ Safe DNA Gel Stain (Thermo Fisher Scientific, Waltham, USA).

### 2.5 DNA sequencing of *cat* and *tet* genes

PCR products that were presumptively positive for *cat* or *tet* genes were purified using the MinElute PCR Purification Kit (Qiagen, Hilden, Germany) and sequenced in one direction using their respective specific gene primer (Supplementary Table S1). Sanger sequencing was performed at The Ramaciotti Centre for Genomics (Sydney, NSW, Australia) using Big Dye Terminator chemistry version 3.1 and an ABI 3730 Capillary Sequencer (Applied Biosystems, Foster City, USA). Sequences were checked for quality using Geneious 2022.1.1 software (Biomatters Limited, Auckland, New Zealand), uploaded to ResFinder 4.1 (available at http://www.genomicepidemiology.org/services/) [49] to confirm the resistance gene variant, and BLASTn searches (https://blast.ncbi.nlm.nih.gov/Blast.cgi) performed to identify sequence matches.

### 2.6 Statistical analyses

Frequencies for each group were summarised using count with percentage. 95% confidence intervals (CI) for individual proportions and the difference in proportions were calculated using score methods (Wilson 1927, Miettinen and Nurminen 1985). Intervals for differences excluding 0 imply significantly different proportions.

## 3 Results

### 3.1 Detection of *cat* and *tet* genes in koala DNA samples

Following 16S rRNA PCR, 485 of 534 koala DNA samples from the different locations were deemed to be PCR competent and were included in this study (Table 1 and individual koala data in Supplementary Data 1).

Overall, DNA sequencing of PCR amplicons confirmed the presence of *cat* and/or *tet* genes in 48 of 485 samples (9.9% of koalas, 95% CI: 7.5% to 12.9%). Six of 485 koalas (1.2%) carried both *tet* and *cat* resistance genes (Table 2).

**Table 2:** Chloramphenicol resistance genes (*cat*) and doxycycline/tetracycline resistance genes (*tet*) detected in DNA from urogenital tract swabs, chlamydial swabs (urogenital and/or conjunctiva) and faecal swabs, from koalas in South Australia (SA). AUS, Australia. mAUS, Mainland Australia. MLR, Mount Lofty Ranges.

Location	Pre-fire/Fire-affected/ Non-fire-affected (Date collected)	No. samples	No. *cat* positive (%)	No. *tet* positive (%)	No. both *cat* and *t*e*t* positive (%)	Total No. *cat* and/or *t*e*t* positive (%)
Cleland, MLR	Pre-fire (2018)	39	2 (5.1)	3 (7.7)	0 (0)	5 (12.8)
Belair, MLR	Pre-fire (2018)	29	0 (0)	1 (3.4)	0 (0)	1 (3.4)
Morialta, MLR	Pre-fire (2018)	23	1 (4.3)	0 (0)	0 (0)	1 (4.3)
	All MLR samples	91	3 (3.3)	4 (4.4)	0 (0)	7 (7.7)
Kangaroo Island	Pre-fire (2014–2017)	46	0 (0.0)	3 (6.5)	0 (0)	3 (6.5)
	Fire (2019–2020)	95	6 (6.3)	14 (14.7)	4 (4.2)	16 (16.8)
	All KI samples	141	6 (4.3)	17 (12.1)	4 (2.8)	19 (13.5)
Mainland AUS	Non-fire (2019–2020)	8	0 (0.0)	1 (12.5)	0 (0)	1 (12.5)
	Fire (2019–2020)	245	7 (2.9)	16 (6.5)	2 (0.8)	21 (8.6)
	All mAUS samples	253	7 (2.8)	17 (6.7)	2 (0.8)	22 (8.7)
Totals	All samples	485	16 (3.3)	38 (7.8)	6 (1.2)	48 (9.9)

Chloramphenicol resistance genes (*cat*) were detected in 3.3% (16 of 485, 95% CI: 2.0% to 5.3%) of koala samples, with the highest frequency (4.3%) in DNA samples from all KI koalas (urogenital swabs; 6 of 141) and the lowest frequency (2.8%) in all mAUS koalas (chlamydial swabs; 7 of 253) (Table 2).

Doxycycline/tetracycline resistance genes (*tet*) were detected in 7.8% (38 of 485, 95% CI: 5.8% to 10.6%) koalas, with the highest frequency (12.1%) in DNA samples from all KI koalas (urogenital swabs; 17 of 141) and the lowest frequency (4.4%) in all MLR koalas (faecal swabs; 4 of 91) (Table 2).

### 3.2 Diversity of *cat* and *tet* genes in koala DNA samples

**Fig. 2:**
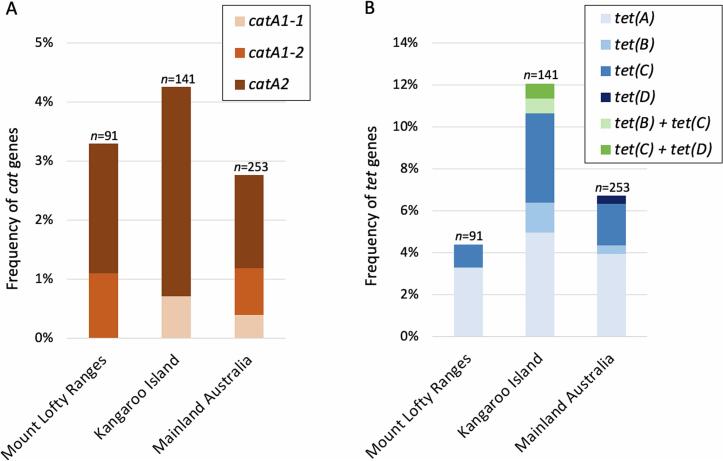
The diversity of chloramphenicol resistance genes (*cat*) and doxycycline/tetracycline resistance genes (*tet*) detected in DNA collected from koalas in Australia; Faecal samples from Mount Lofty Ranges koalas, urogenital swabs from Kangaroo Island koalas and chlamydial swabs (urogenital and conjunctiva) from mainland Australia koalas. **2A.** Frequency of three variants of *cat* genes. **2B.** Frequency of four variants of *tet* genes. All *cat* and *tet* gene variant frequency data is summarised in Supplementary Table S2.

Three variants of *cat* genes, *catA1–1, catA1–2, catA2*, and four variants of *tet* genes, *tet*(A), *tet*(B), *tet*(C) and *tet*(D), were identified in the 485 koala DNA samples, with *tetA* being the most frequent (4.1% of koalas, range 3.3% to 5.0%), followed by *tet*(C) (2.9% of koalas, range 1.1% to 5.7%) and *catA2* (2.3% of koalas, range 1.6% to 3.5%) (Fig. 2A and Fig. 2B). The remaining detected *cat* and *tet* resistance genes were identified in ≤0.8% of the 485 DNA samples (Fig. 2A and Fig. 2B). The *cat* and *tet* gene variants *catA3*, *catA4*, *tet*(E) and *tet*(G) were not detected in any koala DNA samples.

Of the six koalas carrying both *tet* and *cat* genes, the *catA2* gene was detected in all six, four of six also carried one *tet* gene and the remaining two koalas also carried two different *tet* genes (Fig. 2B). Individual koala *cat* and *tet* data is provided in Supplementary Data 1.

BLASTn searches of the two *catA1–1* partial sequences, one from a KI (urogenital swab DNA) and the second from mAUS (chlamydial swab DNA) determined they were 100% and 99.6% sequence matches respectively, to multiple *Proteus mirabilis* chromosomal *cat* genes (top matches were GenBank accessions CP046048 and CP049942 respectively). These two *P. mirabilis* associated *catA1–1* sequences were only 77%–78% homologous to the three *catA1–2* variant partial sequences also detected. BLASTn searches of koala *catA1–2*, *catA2* and all *tet* partial sequences found they were > 99% identity matches to *cat* and *tet* genes from diverse bacterial species, including *Enterobacter*, *Escherichia coli, Klebsiella, Pasteurella, Proteus, Pseudomonas* and *Salmonella,* plus *Chlamydia suis* (*tet*(C) only).

### 3.3 Frequency and gene diversity in fire-affected and non-fire-affected koalas

**Fig. 3:**
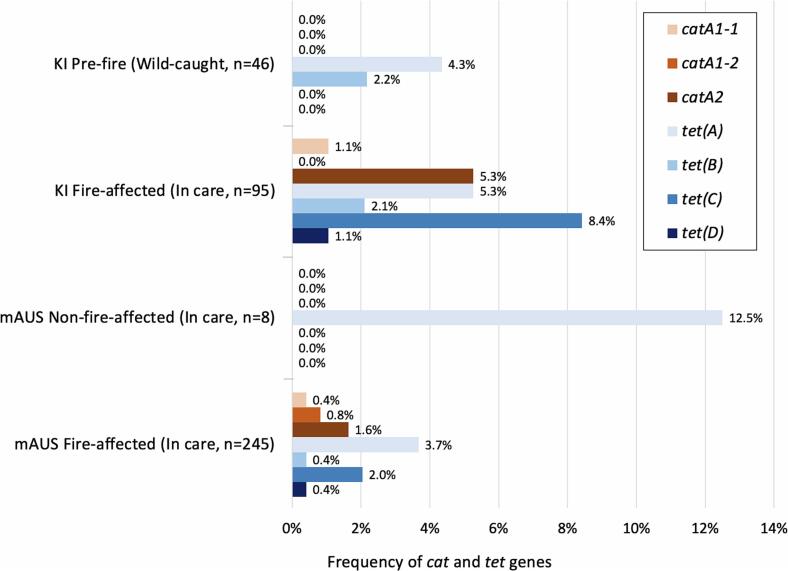
Comparison of chloramphenicol (*cat*) and doxycycline/tetracycline (*tet*) resistance genes frequency in DNA samples from fire-affected and non-fire-affected koalas at two sites in Australia; Urogenital swab DNA from Kangaroo Island (KI) koalas and chlamydial swab (urogenital and conjunctiva) DNA from mainland Australia (mAUS) koalas. All individual koala data is provided in Supplementary Data 1 and all *cat* and *tet* diversity data is summarised in Supplementary Table S2.

The frequency of *cat* and/or *tet* genes detected in KI koalas was higher in samples collected from fire-affected koalas in veterinary care (16.8%, 16 of 95), compared to wild-caught koalas sampled prior to the 2019–2020 wildfires (6.5%, 3 of 46) however the difference was not significant (95% CI for the difference: −2.1% to 20.5%) (Fig. 3). All DNA samples from mAUS were from animals in care, and the frequency of *cat* and/or *tet* genes was lower in samples collected from fire-affected koalas (8.6%, 21 of 245), compared to non-fire-affected koalas (12.5%, 1 of 8), though the difference was not significant (95% CI for the difference: −7.4% to 39.8%) (Fig. 3). However, it should be noted that the mAUS koala non-fire-affected sample was size was small (*n* = 8) compared to the fire-affected sample size (*n* = 245) and only one resistance gene was detected in the non-fire-affected samples (Fig. 3). Of the 22 mAUS DNA samples with *cat* and/or *tet* genes detected, 18 koalas were from SA and four koalas were from an unspecified location.

A low diversity of *cat* and *tet* resistance genes was detected in pre-fire KI DNA samples and non-fire-affected mAUS DNA samples, with only *tet*(A) detected in both sample sets, *tet*(B) in pre-fire KI samples, and no *cat* genes were detected in either sample set (Fig. 3). In contrast, a higher diversity of *cat* and *tet* resistance genes were detected in KI and mAUS DNA samples from fire-affected koalas in veterinary care, with six resistance gene variants detected in the KI fire-affected samples and seven resistance genes detected in the mAUS fire-affected samples (Fig. 3).

The wild-caught MLR koalas were all sampled pre-fire and the overall frequency of *cat* and *tet* genes in faecal DNA was 7.7% (7 of 91) but varied across the three sampled national park/reserve areas; Cleland had the highest frequency (5 of 39, 12.8%), with Belair and Morialta having lower frequencies of 3.4% (1 of 29) and 4.3% (1 of 23) respectively. Cleland also had the highest diversity of resistance genes, with *tet*(A), *tet*(C) and *catA2* detected, whereas only *tet*(A) or *catA1* were detected in Belair and Morialta respectively. All *cat* and *tet* diversity data for MLR koalas is summarised in Supplementary Table S2.

### 3.4 Chlamydial infection status in koalas carrying *cat* and/or *tet* genes

Of the 22 mAUS koalas with *cat* and/or *tet* genes detected in DNA samples, 11 (50%, 95% CI: 31% to 69%) were reported to be *Chlamydia*-positive by diagnostic qPCR performed at the APCAH, with nine carrying one *cat* or *tet* gene and two koalas carrying both *tet* and *cat* genes (Table 3). Of the 11 *Chlamydia*-negative koalas, all carried one *cat* and/or *tet* gene (Table 3). *Chlamydia* diagnostic qPCR testing of KI koalas (*n* = 160) were all reported to be negative, thus all KI koalas carrying *cat* and/or *tet* genes (*n* = 19) were *Chlamydia*-negative. Chlamydial infection status, as determined by qPCR, was unavailable for the MLR koalas, however, physical examination chlamydial scores were provided, with two *cat* or *tet* positive koalas showing clinical signs of chlamydial disease (Supplementary Data 1).

**Table 3:** Chlamydial infection status of mainland Australian koalas carrying *cat* and/or *tet* genes in chlamydial swab DNA samples.

Chlamydial status (qPCR)	*cat* genes carried	*tet* genes carried	No. of koalas
Positive	*catA1–1*	–	1
	*catA1–2*	–	1
	*catA2*	–	2
	–	*tet*(A)	4
	–	*tet*(C)	1
	*catA2*	*tet*(A)	1
	*catA2*	*tet*(D)	1
Negative	*catA1–2*	–	1
	–	*tet*(A)	5
	–	*tet*(B)	1
	–	*tet*(C)	4

### 3.5 Antibiotic administration and carriage of *cat* and *tet* genes

Of the 95 fire-affected KI koalas, veterinary treatments records were available for 59 koalas, and of these, eight koalas (13.6%, 95% CI: 7.0% to 24.5%) received antibiotics prior to the collection of urogenital swabs (Table 4)*.* The frequency of *cat* and/or *tet* genes was significantly higher (37.5%) in DNA samples collected from koalas after receiving antibiotics than in koalas which had not received antibiotics prior to sampling (9.8%, 95% CI for the difference: 19% to 78%) (Table 4). All pre-fire koalas from MLR (*n =* 91) and KI (*n =* 46) were wild-caught and had no history of receiving systemic antibiotic treatment. Antibiotic treatment histories were unavailable for mAUS chlamydial swab koalas.

**Table 4:** Antibiotic administration and carriage of *cat* and *tet* genes in 64 fire-affected Kangaroo Island koalas with available veterinary treatment records.

Antibiotics administered prior to swab collection	*cat* and *tet* genes detected in urogenital swab DNA	No. *cat*/*tet* positive koalas (%)
Amoxicillin +/− clavulanic acid	*tet*(C)	1/8 (12.5%)
	None detected	3/8 (37.5%)
Amoxicillin +/− clavulanic acid and enrofloxacin	*tet*(A)	1/8 (12.5%)
	None detected	2/8 (25.0%)
Amoxicillin and enrofloxacin	*catA2, tet*(C) and *tet*(D)	1/8 (12.5%)
Total - Received antibiotics	Total *cat/tet* positive	3/8 (37.5%)
	Total *cat/tet* negative	5/8 (62.5%)
No antibiotics administered	*catA2* and *tet*(C) *tet*(A) *tet*(C) None detected	1/51 (1.96%) 2/51 (3.9%) 2/51 (3.9%) 46/51 (90.2%)
Total - No antibiotics	Total *cat/tet* positive	5/51 (9.8%)
	Total *cat/tet* negative	46/51 (90.2%)

### 3.6 Frequency of *cat* and *tet* genes in female and male koalas

Of the 91 MLR faecal samples, 68 were collected from female koalas and 23 from male koalas. The frequency of *cat* and *tet* genes in male MLR koalas was significantly higher (17.4%) compared to female MLR koalas (4.4%), 95% CI for the difference: (0.3%–33.3%)*.* From a total of 95 fire-affected koalas on KI, 32 were female, 36 were male and the sex of 27 koalas was unknown. Of the 68 fire-affected koalas on KI with a known sex, the frequency of *cat* and *tet* genes in DNA samples was slightly higher for males (16.7%) compared to females (12.5%), 95% CI for the difference (−14.1% to 21.8%)*.* All 46 pre-fire koalas on KI were female and the frequency of *cat* and *tet* genes in samples was 6.5%**.** The sex of mAUS koalas was unavailable.

## 4 Discussion

This study is the first to report the presence of genes conferring resistance against the two anti-chlamydial antibiotics of choice in koalas, namely chloramphenicol and doxycycline [18,[21], [22], [23], [24]], in the urogenital/ocular and faecal microbiomes of koalas. Carriage of diverse *cat* and *tet* genes occurred in both *Chlamydia*-free and *Chlamydia*-positive koalas, and in the absence of antibiotic administration. Fire-affected koalas in care carried the highest frequencies and diversity of *cat* and/or *tet* genes compared to pre-fire wild koalas, indicating increased exposure to and acquisition of antimicrobial resistant bacteria by fire-affected koalas in care. Additional factors, including physiological stress, exposure to a different ecological environment and dietary changes, may also contribute to microbiome shifts and increased carriage of antimicrobial resistant bacteria by koalas entering care [50,51]. Increased carriage of antimicrobial resistance genes by wildlife in care or captivity, compared to their free-living counterparts, has previously been reported in Australian wildlife including, grey-headed flying foxes (*Pteropus poliocephalus*) [28], brush-tail rock wallabies (*Petrogale penicillata*) [52], Australian sea lions (*Neophoca cinerea*) [53] and birds [10].

Chloramphenicol was not administered to koalas sampled on KI and doxycycline was administered to only one koala after urogenital swab sampling. However, the carriage of *cat* and/or *tet* genes was higher in koalas that received other antibiotics prior to urogenital swab sampling compared to koalas that did not receive antibiotic treatment. The presence of resistant genes to antibiotics that were not administered suggests that antibiotic treatment of koalas may have co-selected for bacteria which carry multiple types of resistance genes [29].

The bacterial species carrying the chloramphenicol and doxycycline resistance genes in koala microbiomes were not identified as part of this study. The detection of diverse *cat* and *tet* gene variants in both *Chlamydia*-free and *Chlamydia*-positive koalas, indicates that resistance genes were most likely carried by non-chlamydial bacterial species. The *catA1–1* gene detected in two koalas was a sequence match to the distinct *Proteus mirabilis* chromosomal *cat* gene variant [54], strongly suggesting these two koalas carried chloramphenicol resistant *P. mirabilis* strains in their urogenital microbiomes. The remaining *cat* and *tet* genes detected in koala microbiomes (*catA1–2, catA2, tet*(A), *tet*(B), *tet*(C) and *tet*(D)) have been previously identified in a diverse range of gram-negative bacterial species, including *Citrobacter, Enterobacter, Escherichia, Klebsiella, Proteus, Pseudomonas* and *Salmonella* [32] and the *tet*(C) gene in *Chlamydia suis* [34].

Antibiotic administration, particularly when administered orally, has been shown to induce shifts in the composition of the intestinal microbiome and to select for resistant bacterial strains [55,56]. Although oral administration of antibiotics is avoided in koalas, administration of antibiotics via subcutaneous, intramuscular, and intravenous injection can still alter gut microbiome composition [20,57,58]. Given that koalas are inherently susceptible to antibiotic induced gastrointestinal dysbiosis [20], chloramphenicol or doxycycline administration to koalas carrying resistant bacterial strains has the potential to exacerbate or induce dysbiosis by increasing the abundance of resistant bacteria in the gut microbiome [59].

Selective pressure associated with antibiotic treatment also has the potential to unintentionally select for resistant populations of opportunistic bacterial pathogens in the faecal microbiome [55,56], including strains capable of establishing intestinal and urinary tract infections [60,61]. The *tet* and *cat* genes detected in koala microbiomes are carried by diverse gram-negative bacterial strains, including opportunistic pathogens [32]. In koalas with antibiotic induced gastrointestinal dysbiosis, the loss of mucobacterial lining of caecum and proximal colon makes the intestinal wall extremely susceptible to secondary bacterial infections and ulceration [19], which may be exacerbated if resistant opportunistic pathogens are present. Multiple species of opportunistic pathogens have been identified in the urogenital tract of koalas with chlamydial disease, including gram-negatives (*Proteus*, *Escherichia coli, Pasteurella and Klebsiella oxytoca*) and gram-positives (*Bacillus, Staphylococcus* and *Streptococcus*) [[62], [63], [64], [65]]. Concerningly, one of the two koalas potentially carrying chloramphenicol resistant *P. mirabilis* in its urogenital microbiome was also *Chlamydia*-positive.

Antibiotic administration to individuals co-infected with antimicrobial resistant bacteria and non-resistant bacterial pathogens provides an opportunity for horizontal transfer of resistance genes between bacterial species, particularly when dysbiosis occurs, potentially driving the emergence of new antimicrobial resistant bacterial pathogens [56,60]. The use of antibiotics in farmed pigs has acted as a selective pressure to promote horizontal gene transfer of *tet*(C) from a non-chlamydial bacterial strain to *C. suis* [34] and between different *C. suis* strains [48]. In this study, we observed concurrent *C. pecorum* infection and carriage of *cat* and/or *tet* genes in 11 koalas. In these cases, there is the potential for *C. pecorum* to acquire *cat* or *tet* genes via horizontal transfer [66], consequently reducing chloramphenicol or doxycycline efficacy and resulting in treatment failure [67]. To date, there has been only one published report which found no evidence of chloramphenicol resistance in *C. pecorum* isolates from koalas [68] and there are no published reports of susceptibility of *C. pecorum* to doxycycline. Further research is warranted to determine if antimicrobial resistant strains of *C. pecorum* have emerged in koalas.

This study targeted the detection of the most frequently observed chloramphenicol and doxycycline resistance genes (*cat* and *tet*) in gram-negative bacteria [32]. Koala faecal and urogenital microbiomes carry diverse species of both gram-negative and gram-positive bacteria [20,[69], [70], [71]], which potentially carry an extensive range of additional chloramphenicol and doxycycline resistance genes [32]. Thus, the frequency of *cat* and *tet* resistance genes reported in this study is likely to be an under-estimation of the prevalence of chloramphenicol and doxycycline resistance in koala microbiomes.

Given that this study focused on koalas from SA, it would be prudent to perform a comparative study in koalas from Queensland and New South Wales, where chlamydial disease is a bigger threat to koala conservation [12,25]. The ongoing threat of wildfires also makes it imperative to understand the role of wildfires and antibiotic use as drivers of AMR ecology in koalas across their geographical range in Australia. Additional research is also required to elucidate the bacterial carriers of resistance genes in koala microbiomes, identify resistant opportunistic bacterial pathogens and ensure successful treatment for chlamydial disease in koalas. The emergence and spread of antibiotic resistant *C. pecorum* strains has the potential to increase the incidence of non-resolving chlamydial disease and treatment failure. Carriage of other chloramphenicol and doxycycline resistant bacteria may also play a role in negative treatment outcomes of koalas receiving antibiotics for chlamydial disease, specifically, increase the risk of fatal gastrointestinal dysbiosis events occurring and induce or exacerbate secondary bacterial infections.

The presence of chloramphenicol and doxycycline resistant genes in koala microbiomes may increase the already significant threat of chlamydial disease on Australia's koalas. Additionally, the data from this study indicates that koalas which are fire-affected and require veterinary care, and notably those that receive antibiotic treatment, have an increased risk of acquiring chloramphenicol and doxycycline resistant determinants and/or bacteria. These findings highlight an additional conservation threat for koalas and reinforce the need for judicious use of anti-chlamydial antibiotics and adherence to antimicrobial prescribing guidelines when treating fire-affected koalas.

The *cat* and *tet* genes detected in koala microbiomes are frequently associated with anthropogenic gram-negative pathogens [32], highlighting the interconnected relationship of AMR between humans and wildlife, and the One Health significance. The integration of antimicrobial resistance traits into koala microbiomes and identified potential negative health impacts for koalas undergoing treatment for chlamydial disease, is also being exacerbated by anthropogenic threats, including habitat loss and wildfires [25]. This project demonstrates the importance of considering threats posed by AMR to wildlife health and the multiple anthropogenic impacts nested within the One Health framework, that are driving these AMR threats. There is a need for greater understanding of AMR ecology in wildlife and a holistic intersectoral One Health approach to AMR that considers wildlife health and conservation, and to ensure a true multidisciplinary AMR One Health approach.

## Funding

This study was funded by the 10.13039/100001250Morris Animal Foundation (Grant No. D21ZO-507 to Michelle Power and Grant No. D21ZO-506 to Natasha Speight).

## Animal ethics

Mounty Lofty Ranges koala rectal swab sample collection was approved by The University of Adelaide Animal Ethics Committee approval number S-2018-022 and the South Australian Department for Environment and Water (DEW) Scientific Research permit number Y26054-7. Kangaroo Island pre-fire koala urogenital swab sample collection was approved by The University of Adelaide Animal Ethics Committee approval number S-2013-198 and the South Australian Department for Environment and Water (DEW) Scientific Research permit number Y26054-6. The acquisition of archived chlamydial disease diagnostic samples collected from koalas in veterinary care on Kangaroo Island and in mainland Australia was approved under the Macquarie University Animal Ethics Committee (ARA permit Reference No. 2020/027-2) permitting the collection samples from koalas in care.

## CRediT authorship contribution statement

**Fiona K. McDougall:** Conceptualization, Data curation, Formal analysis, Investigation, Methodology, Project administration, Validation, Visualization, Writing – original draft, Writing – review & editing. **Wayne S.J. Boardman:** Conceptualization, Data curation, Investigation, Resources, Writing – review & editing. **Natasha Speight:** Data curation, Funding acquisition, Investigation, Methodology, Resources, Writing – review & editing. **Tamsyn Stephenson:** Data curation, Formal analysis, Investigation, Methodology, Resources, Writing – review & editing. **Oliver Funnell:** Data curation, Investigation, Resources, Writing – review & editing. **Ian Smith:** Data curation, Investigation, Resources, Writing – review & editing. **Petra L. Graham:** Formal analysis, Writing – review & editing. **Michelle L. Power:** Conceptualization, Funding acquisition, Investigation, Methodology, Project administration, Resources, Supervision, Writing – review & editing.

## Declaration of Competing Interest

The authors declare the following financial interests/personal relationships which may be considered as potential competing interests:

Michelle Power reports financial support was provided by 10.13039/100001250Morris Animal Foundation. Natasha Speight reports financial support was provided by 10.13039/100001250Morris Animal Foundation.

## Data Availability

All data for individual koalas is provided in a supplementary file (Supplementary Data 1) and additional PCR methods and data tables are provided in a supplementary tables file (Supplementary Tables).

## Appendix A Supplementary data

Supplementary material 1 Image 1

Supplementary material 2 Image 2

## References

[1] Vittecoq M., Godreuil S., Prugnolle F., Durand P., Brazier L., Renaud N., Arnal A., Aberkane S., Jean-Pierre H., Gauthier-Clerc M. (2016). Antimicrobial resistance in wildlife. J. Appl. Ecol..

[2] Collignon P.J., McEwen S.A. (2019). One health—its importance in helping to better control antimicrobial resistance. Trop. Med. Infect. Dis..

[3] Carroll D., Wang J., Fanning S., McMahon B.J. (2015). Antimicrobial resistance in wildlife: implications for public health, Zoonoses Publ. Health..

[4] Stephen C., Wilcox A., Sine S., Provencher J. (2023). A reimagined one health framework for wildlife conservation. Res. Dir.: One Health.

[5] Fulham M., McDougall F., Power M., McIntosh R.R., Gray R. (2022). Carriage of antibiotic resistant bacteria in endangered and declining Australian pinniped pups. PLoS One.

[6] McDougall F., Boardman W., Power M. (2022). High prevalence of beta-lactam resistant *Escherichia coli* in South Australian grey-headed flying fox pups (*Pteropus poliocephalus*). Microorg..

[7] Smith H.G., Bean D.C., Clarke R.H., Loyn R., Larkins J.A., Hassell C., Greenhill A.R. (2022). Presence and antimicrobial resistance profiles of *Escherichia coli, Enterococcus* spp. and *Salmonella* sp. in 12 species of Australian shorebirds and terns. Zoonoses Public Health.

[8] McDougall F.K., Boardman W.S., Power M.L. (2021). Characterization of beta-lactam-resistant *Escherichia coli* from Australian fruit bats indicates anthropogenic origins. Microb. Genomics.

[9] Mukerji S., Stegger M., Truswell A.V., Laird T., Jordan D., Abraham R.J., Harb A., Barton M., O’Dea M., Abraham S. (2019). Resistance to critically important antimicrobials in Australian silver gulls (*Chroicocephalus novaehollandiae*) and evidence of anthropogenic origins. J. Antimicrob. Chemother..

[10] Blyton M.D., Pi H., Vangchhia B., Abraham S., Trott D.J., Johnson J.R., Gordon D.M. (2015). Genetic structure and antimicrobial resistance of *Escherichia coli* and cryptic clades in birds with diverse human associations. Appl. Environ. Microbiol..

[11] Dolejska M., Masarikova M., Dobiasova H., Jamborova I., Karpiskova R., Havlicek M., Carlile N., Priddel D., Cizek A., Literak I. (2015). High prevalence of *Salmonella* and IMP-4-producing Enterobacteriaceae in the silver gull on Five Islands, Australia. J. Antimicrob. Chemother..

[12] Quigley B.L., Timms P. (2020). Helping koalas battle disease–recent advances in *Chlamydia* and koala retrovirus (KoRV) disease understanding and treatment in koalas. FEMS Microbiol. Rev..

[13] Vitali S.D., Reiss A.E., Jakob-Hoff R.M., Stephenson T.L., Holz P.H., Higgins D.P. (2022).

[14] Jelocnik M., Gillett A., Hanger J., Polkinghorne A., Vogelnest L., Portas T. (2019). Current Therapy in Medicine of Australian Mammals.

[15] Fabijan J., Caraguel C., Jelocnik M., Polkinghorne A., Boardman W.S., Nishimoto E., Johnsson G., Molsher R., Woolford L., Timms P. (2019). *Chlamydia pecorum* prevalence in South Australian koala (*Phascolarctos cinereus*) populations: identification and modelling of a population free from infection. Sci. Rep..

[16] Speight K., Hicks P., Graham C., Boardman W., Breed W., Manthorpe E., Funnell O., Woolford L. (2018). Necropsy findings of koalas from the Mount Lofty Ranges population in South Australia. Aust. Vet. J..

[17] Speight K.N., Polkinghorne A., Penn R., Boardman W., Timms P., Fraser T., Johnson K., Faull R., Bate S., Woolford L. (2016). Prevalence and pathologic features of *Chlamydia pecorum* infections in South Australian koalas (*Phascolarctos cinereus*). J. Wildl. Dis..

[18] Robbins A., Loader J., Timms P., Hanger J. (2018). Optimising the short and long-term clinical outcomes for koalas (*Phascolarctos cinereus*) during treatment for chlamydial infection and disease. PLoS One.

[19] Gillett A., Hanger J., Vogelnest L., Portas T. (2019). Current Therapy in Medicine of Australian Mammals.

[20] Dahlhausen K.E., Doroud L., Firl A.J., Polkinghorne A., Eisen J.A. (2018). Characterization of shifts of koala (*Phascolarctos cinereus*) intestinal microbial communities associated with antibiotic treatment. PeerJ..

[21] Booth R., Nyari S. (2020). Clinical comparison of five anti-chlamydial antibiotics in koalas (*Phascolarctos cinereus*). PLoS One.

[22] Chen C.J., Gillett A., Booth R., Kimble B., Govendir M. (2022). Pharmacokinetic profile of doxycycline in koala plasma after weekly subcutaneous injections for the treatment of chlamydiosis. Animals..

[23] Black L., McLachlan A., Griffith J., Higgins D., Gillett A., Krockenberger M., Govendir M. (2013). Pharmacokinetics of chloramphenicol following administration of intravenous and subcutaneous chloramphenicol sodium succinate, and subcutaneous chloramphenicol, to koalas (*Phascolarctos cinereus*). J. Vet. Pharmacol. Ther..

[24] Govendir M., Hanger J., Loader J., Kimble B., Griffith J., Black L., Krockenberger M., Higgins D. (2012). Plasma concentrations of chloramphenicol after subcutaneous administration to koalas (*Phascolarctos cinereus*) with chlamydiosis. J. Vet. Pharmacol. Ther..

[25] McAlpine C., Lunney D., Melzer A., Menkhorst P., Phillips S., Phalen D., Ellis W., Foley W., Baxter G., De Villiers D. (2015). Conserving koalas: a review of the contrasting regional trends, outlooks and policy challenges. Biol. Conserv..

[26] Lunney D., Cope H., Sonawane I., Stalenberg E., Haering R. (2022). An analysis of the long-term trends in the records of friends of the koala in north-east New South Wales: I. Cause and fate of koalas admitted for rehabilitation (1989–2020). Pac. Conserv. Biol..

[27] Kerlin D.H., Grogan L.F., McCallum H.I. (2022). Insights and inferences on koala conservation from records of koalas arriving to care in south-east Queensland. Wildl. Res..

[28] McDougall F., Boardman W., Gillings M., Power M. (2019). Bats as reservoirs of antibiotic resistance determinants: a survey of class 1 integrons in grey-headed flying foxes (*Pteropus poliocephalus*). Infect. Genet. Evol..

[29] Ishihara K., Hosokawa Y., Makita K., Noda J., Ueno H., Muramatsu Y., Mukai T., Yamamoto H., Ito M., Tamura Y. (2012). Factors associated with antimicrobial-resistant *Escherichia coli* in zoo animals. Res. Vet. Sci..

[30] Kinjo T., Minamoto N., Sugiyama M., Sugiyama Y. (1992). Comparison of antimicrobial resistant *Escherichia coli* in wild and captive Japanese serows. J. Vet. Med. Sci..

[31] Ahmed A.M., Motoi Y., Sato M., Maruyama A., Watanabe H., Fukumoto Y., Shimamoto T. (2007). Zoo animals as reservoirs of gram-negative bacteria harboring integrons and antimicrobial resistance genes. Appl. Environ. Microbiol..

[32] Roberts M.C., Schwarz S., Mayers D.L., Sobel J.D., Ouellette M., Kaye K.S., Marchaim D. (2017). Antimicrobial Drug Resistance: Mechanisms of Drug Resistance.

[33] Sandoz K.M., Rockey D.D. (2010). Antibiotic resistance in *Chlamydiae*. Future Microbiol..

[34] Dugan J., Rockey D.D., Jones L., Andersen A.A. (2004). Tetracycline resistance in *Chlamydia suis* mediated by genomic islands inserted into the chlamydial inv-like gene. Antimicrob. Agents Chemother..

[35] Wintle B.A., Legge S., Woinarski J.C. (2020). After the megafires: what next for Australian wildlife?. Trends Ecol. Evol..

[36] Government of South Australia (2020). Independent Review into South Australia's 2019–20 Bushfire Season. https://safecom-files-v8.s3.amazonaws.com/current/docs/Independent%2520Review%2520into%2520SA%2527s%25202019-20%2520Bushfire%2520Season%2520-%2520Web%2520Upload.pdf.

[37] Khan S.J. (2021). Ecological consequences of Australian “Black summer”(2019–20) fires: a synthesis of Australian commonwealth government report findings. Integr. Environ. Assess. Manag..

[38] Dunstan E., Funnell O., McLelland J., Stoeckeler F., Nishimoto E., Mitchell D., Mitchell S., McLelland D.J., Kalvas J., Johnson L. (2021). An analysis of demographic and triage assessment findings in bushfire-affected koalas (*Phascolarctos cinereus*) on Kangaroo Island, South Australia, 2019–2020. Animals..

[39] Parrott M.L., Wicker L.V., Lamont A., Banks C., Lang M., Lynch M., McMeekin B., Miller K.A., Ryan F., Selwood K.E. (2021). Emergency response to Australia’s black summer 2019–2020: the role of a zoo-based conservation organisation in wildlife triage, rescue, and resilience for the future. Animals..

[40] Lunney D., Cope H., Sonawane I., Haering R. (2022). A state-wide picture of koala rescue and rehabilitation in New South Wales during the 2019–2020 bushfires. Aust. Zool..

[41] Australian Government (1999). https://www.environment.gov.au/cgi-bin/sprat/public/publicspecies.pl?taxon_id=85104.

[42] Lane D., Stackebrandt E., Goodfellow M. (1991). Nucleic Acid Techniques in Bacterial Systematics.

[43] Hulse L.S., Hickey D., Mitchell J.M., Beagley K.W., Ellis W., Johnston S.D. (2018). Development and application of two multiplex real-time PCR assays for detection and speciation of bacterial pathogens in the koala. J. Vet. Diagn. Investig..

[44] Stephenson T. (2021).

[45] Robertson T., Bibby S., O’Rourke D., Belfiore T., Lambie H., Noormohammadi A. (2009). Characterization of *Chlamydiaceae* species using PCR and high resolution melt curve analysis of the 16S rRNA gene. J. Appl. Microbiol..

[46] Yoo M.H., Huh M.-D., Kim E.-H., Lee H.-H., Do Jeong H. (2003). Characterization of chloramphenicol acetyltransferase gene by multiplex polymerase chain reaction in multidrug-resistant strains isolated from aquatic environments. Aquaculture..

[47] Jun L.J., Jeong J.B., Huh M.-D., Chung J.-K., Choi D.-L., Lee C.-H., Do Jeong H. (2004). Detection of tetracycline-resistance determinants by multiplex polymerase chain reaction in Edwardsiella tarda isolated from fish farms in Korea. Aquaculture..

[48] Marti H., Kim H., Joseph S.J., Dojiri S., Read T.D., Dean D. (2017). *Tet* (C) gene transfer between *Chlamydia suis* strains occurs by homologous recombination after co-infection: implications for spread of tetracycline-resistance among *Chlamydiaceae*. Front. Microbiol..

[49] Bortolaia V., Kaas R.S., Ruppe E., Roberts M.C., Schwarz S., Cattoir V., Philippon A., Allesoe R.L., Rebelo A.R., Florensa A.F. (2020). ResFinder 4.0 for predictions of phenotypes from genotypes. J. Antimicrob. Chemother..

[50] Narayan E., Vanderneut T. (2019). Physiological stress in rescued wild koalas are influenced by habitat demographics, environmental stressors, and clinical intervention. Front. Endocrinol. (Lausanne).

[51] Dallas J.W., Warne R.W. (2023). Captivity and animal microbiomes: potential roles of microbiota for influencing animal conservation. Microb. Ecol..

[52] Power M.L., Emery S., Gillings M.R. (2013). Into the wild: dissemination of antibiotic resistance determinants via a species recovery program. PLoS One.

[53] Delport T.C., Harcourt R.G., Beaumont L.J., Webster K.N., Power M.L. (2015). Molecular detection of antibiotic-resistance determinants in *Escherichia coli* isolated from the endangered Australian sea lion (*Neophoca cinerea*). J. Wildl. Dis..

[54] Charles I., Keyte J., Shaw W. (1985). Nucleotide sequence analysis of the *cat* gene of *Proteus mirabilis*: comparison with the type I (Tn*9*) *cat* gene. J. Bacteriol..

[55] Modi S.R., Collins J.J., Relman D.A. (2014). Antibiotics and the gut microbiota. J. Clin. Invest..

[56] Stecher B., Maier L., Hardt W.-D. (2013). 'Blooming' in the gut: how dysbiosis might contribute to pathogen evolution. Nat. Rev. Microbiol..

[57] Liu J., Deng X.-C., Li X.-Y., Yang Z.-B., Zhang G.-Y., Chen T.-T. (2020). Intramuscular injection of tetracycline decreased gut microbial diversity in mouse. Mamm. Genome.

[58] Zhang L., Huang Y., Zhou Y., Buckley T., Wang H.H. (2013). Antibiotic administration routes significantly influence the levels of antibiotic resistance in gut microbiota. Antimicrob. Agents Chemother..

[59] Dawsom K.A., Langlois B.E., Stahly T.S., Cromwell G.L. (1984). Antibiotic resistance in anaerobic and coliform bacteria from the intestinal tract of swine fed therapeutic and subtherapeutic concentrations of chlortetracycline. J. Anim. Sci..

[60] Raplee I., Walker L., Xu L., Surathu A., Chockalingam A., Stewart S., Han X., Rouse R., Li Z. (2021). Emergence of nosocomial associated opportunistic pathogens in the gut microbiome after antibiotic treatment, Antimicrob. Resist. Infect. Control..

[61] Hillier S., Roberts Z., Dunstan F., Butler C., Howard A., Palmer S. (2007). Prior antibiotics and risk of antibiotic-resistant community-acquired urinary tract infection: a case–control study. J. Antimicrob. Chemother..

[62] Higgins D., Hemsley S., Canfield P. (2005). Immuno-histochemical demonstration of the role of *Chlamydiaceae* in renal, uterine and salpingeal disease of the koala, and demonstration of *Chlamydiaceae* in novel sites. J. Comp. Pathol..

[63] Obendorf D.L. (1983). Causes of mortality and morbidity of wild koalas, *Phascolarctos cinereus* (Goldfuss), in Victoria, Australia. J. Wildl. Dis..

[64] Weigler B.J., Girjes A.A., White N.A., Kunst N.D., Carrick F.N., Lavin M.F. (1988). Aspects of the epidemiology of *Chlamydia psittaci* infection in a population of koalas (*Phascolarctos cinereus*) in southeastern Queensland, Australia. J. Wildl. Dis..

[65] Canfield P., Oxenford C., Love D., Dickens R. (1983). Pyometra and pyovagina in koalas. Aust. Vet. J..

[66] Marti H., Suchland R.J., Rockey D.D. (2022). The impact of lateral gene transfer in *Chlamydia*. Front. Cell. Infect. Microbiol..

[67] Borel N., Leonard C., Slade J., Schoborg R.V. (2016). Chlamydial antibiotic resistance and treatment failure in veterinary and human medicine. Curr. Clin. Microb. Rep..

[68] Black L., Higgins D., Govendir M. (2015). In vitro activity of chloramphenicol, florfenicol and enrofloxacin against *Chlamydia pecorum* isolated from koalas (*Phascolarctos cinereus*). Aust. Vet. J..

[69] Barker C.J., Gillett A., Polkinghorne A., Timms P. (2013). Investigation of the koala (*Phascolarctos cinereus*) hindgut microbiome via 16S pyrosequencing. Vet. Microbiol..

[70] Legione A.R., Amery-Gale J., Lynch M., Haynes L., Gilkerson J.R., Sansom F.M., Devlin J.M. (2018). Variation in the microbiome of the urogenital tract of *Chlamydia*-free female koalas (*Phascolarctos cinereus*) with and without ‘wet bottom’. PLoS One.

[71] Vidgen M.E., Hanger J., Timms P. (2017). Microbiota composition of the koala (*Phascolarctos cinereus*) ocular and urogenital sites, and their association with *Chlamydia* infection and disease. Sci. Rep..

