# Supplementary material for: Carriage of antibiotic resistance genes to treatments for chlamydial disease in koalas (Phascolarctos cinereus): A comparison of occurrence before and during catastrophic wildfires
Source: One Health. 2023 Nov 10;17:100652. doi: 10.1016/j.onehlt.2023.100652 (PMC10665209; doi:10.1016/j.onehlt.2023.100652)
Supplement: Supplementary file 1 — Supplementary material 1 [file mmc1.pdf]

Supplementary Tables

Supplementary Table S1. Primers used in *cat* and *tet* multiplex PCRs.

**Primer target**	**Primer**	**Primer direction**	**Product size bp**	**Primer sequence (5′-to 3′)**	**Reference**
catA1	CAT-1	Forward	349	GGTGATATGGGATAGTGTT	[45]
catA2	CAT-2	Forward	567	GATTGACCTGAATACCTGGAA	[45]
catA3	CAT-3	Forward	275	CCATACTCATCCGATATTGA	[45]
catA4	CAT-4	Forward	451	CCGGTAAAGCGAAATTGTAT	[45]
cat (All)	CAT-R	Reverse	-	CCATCACATACTGCATGATG	[45]
tet (All)	TETF	Forward	-	GCGCTNTATGCGTTGATGCA	[46]
tet(A)	TAR	Reverse	387	ACAGCCCGTCAGGAAATT	[46]
tet(B)	TBR	Reverse	171	TGAAAGCAAACGGCCTAA	[46]
tet(C)	TCR	Reverse	631	CGTGCAAGATTCCGAATA	[46]
tet(D)	TDR	Reverse	484	CCAGAGGTTTAAGCAGTGT	[46]
tet(E)	TER	Reverse	246	ATGTGTCCTGGATTCCT	[46]
tet(G)	TGR	Reverse	803	ATGCCAACACCCCCGGCG	[46]

Supplementary Table S2. Frequency of specific chloramphenicol resistance gene variants (*catA, catA1, catA2*) and doxycycline/tetracycline resistance gene variants (*tet*(A), *tet*(B), *tet*(C) and *tet*(D)) detected in DNA from urogenital tract (UGT) swabs, chlamydial swabs (UGT and/or conjunctiva) and faecal swabs, from koalas in South Australia (SA). KI, Kangaroo Island. MLR, Mount Lofty Ranges.

**Location**	**Pre-fire/Fire-affected/ Non-fire-affected (Date collected)**	**Sample type**	**Wild/ In care**	**No. samples**	**No. *catA1-1* (%)**	**No. *catA1-2* (%)**	**No. *catA2* (%)**	**No. *tet*(A) (%)**	**No. *tet*(B) (%)**	**No. *tet*(C) (%)**	**No. *tet*(D) (%)**
Cleland, MLR	Pre-fire (2018)	Faecal swab	Wild	39	0 (0.0%)	0 (0.0%)	2 (5.1%)	2 (5.1%)	0 (0.0%)	1 (2.6%)	0 (0.0%)
Belair, MLR	Pre-fire (2018)	Faecal swab	Wild	29	0 (0.0%)	0 (0.0%)	0 (0.0%)	1 (3.4%)	0 (0.0%)	0 (0.0%)	0 (0.0%)
Morialta, MLR	Pre-fire (2018)	Faecal swab	Wild	23	0 (0.0%)	1 (4.3%)	0 (0.0%)	0 (0.0%)	0 (0.0%)	0 (0.0%)	0 (0.0%)
	All MLR samples			91	0 (0.0%)	1 (1.1%)	2 (2.2%)	3 (3.3%)	0 (0.0%)	1 (1.1%)	0 (0.0%)
Kangaroo Island	Pre-fire (2014-2017)	Urogenital swab	Wild	46	0 (0.0%)	0 (0.0%)	0 (0.0%)	2 (4.3%)	1 (2.2%)	0 (0.0%)	0 (0.0%)
Kangaroo Island	Fire (2019-2020)	Urogenital swab	In care	95	1 (1.1%)	0 (0.0%)	5 (5.3%)	5 (5.3%)	2 (2.1%)	8 (8.4%)	1 (1.1%)
	All KI samples			141	1 (0.7%)	0 (0.0%)	5 (3.5%)	7 (5.0%)	3 (2.1%)	8 (5.7%)	1 (0.7%)
Mainland SA	Non-fire (2019-2020)	Chlamydial swab	In care	8	0 (0.0%)	0 (0.0%)	0 (0.0%)	1 (12.5%)	0 (0.0%)	0 (0.0%)	0 (0.0%)
Mainland SA	Fire (2019-2020)	Chlamydial swab	In care	245	1 (0.4%)	2 (0.8%)	4 (1.6%)	9 (3.7%)	1 (0.4%)	5 (2.0%)	1 (0.4%)
	All Mainland SA samples			253	1 (0.4%)	2 (0.8%)	4 (1.6%)	10 (4.0%)	1 (0.4%)	5 (2.0%)	1 (0.4%)
Totals	All samples			485	2 (0.4%)	3 (0.6%)	11 (2.3%)	20 (4.1%)	4 (0.8%)	14 (2.9%)	2 (0.4%)
